# Laparoscopic liver resection of segment seven: A case report and review of surgical techniques

**DOI:** 10.1016/j.ijscr.2020.06.107

**Published:** 2020-07-10

**Authors:** Kosei Takagi, Takashi Kuise, Yuzo Umeda, Ryuichi Yoshida, Fuminori Teraishi, Takahito Yagi, Toshiyoshi Fujiwara

**Affiliations:** Department of Gastroenterological Surgery, Okayama University Graduate School of Medicine, Dentistry, and Pharmaceutical Sciences, Okayama, Japan

**Keywords:** Laparoscopic, Liver, Segment seven

## Abstract

**Introduction:**

Laparoscopic liver resection of segment seven (LLR-S7) is a technically challenging procedure due to its anatomical location and difficult accessibility. Herein, we present our experience with LLR-S7, and demonstrate a literature review regarding surgical techniques.

**Presentation of case:**

A 28-year-old female was diagnosed with rectosigmoid cancer and synchronous liver metastases at the segment three (S3) and S7, which were treated with laparoscopic procedure. After the completely mobilization of the right lobe, the Glissonean pedicle of S7 (G7) was intrahepatically transected. The right hepatic vein was exposed to identify the venous branch of S7 (V7). Finally the liver parenchyma between RHV and dissection line was divided.

**Discussion:**

Various laparoscopic approaches for S7 have been reported including the Glissonian approach from the hilum, the intrahepatic Glissonean approach, the caudate lobe first approach, and the lateral approach from intercostal ports. To perform LLR-S7 safely, it is important to understand the advantage of each technique including the trocar placement and approaches to S7 by laparoscopy.

**Conclusion:**

We present our experience of LLR-S7 for the tumor located at the top of S7, successfully performed with the intrahepatic Glissonean approach. LLR-S7 can be performed safely with advanced laparoscopic techniques and sufficient knowledge on various approaches for S7.

## Highlights

• Laparoscopic liver resection of segment 7 (LLR-S7) is technically challenging procedure.
• Various laparoscopic approaches for S7 have been reported.
• Sufficient knowledge on various approaches for S7 is needed to perform LLR-S7 safely.
• Our experience and a literature review on surgical techniques of LLR-S7 are demonstrated.

## 1 Introduction

Laparoscopic liver resection of segment seven (LLR-S7) is a technically challenging procedure due to its anatomical location in proximity to the right hepatic vein (RHV) and inferior vena cava (IVC) [1,2]. Proper exposure of the surgical view and accessibility by surgical instruments have been reported to be difficult in LLR-S7 [3]. As several approaches for LLR-S7 have been demonstrated to perform the procedure safely, an important issue is to understand each characteristic of different techniques. However, no study has performed a literature review focusing on different approaches to S7 by laparoscopy. The aim of this study is to present our experience with LLR-S7 for the tumor located at the top of S7, and demonstrate a literature review with special regards to surgical techniques. The study is presented in accordance with the SCARE Guidelines [4].

## 2 Presentation of case

**Fig. 1:**
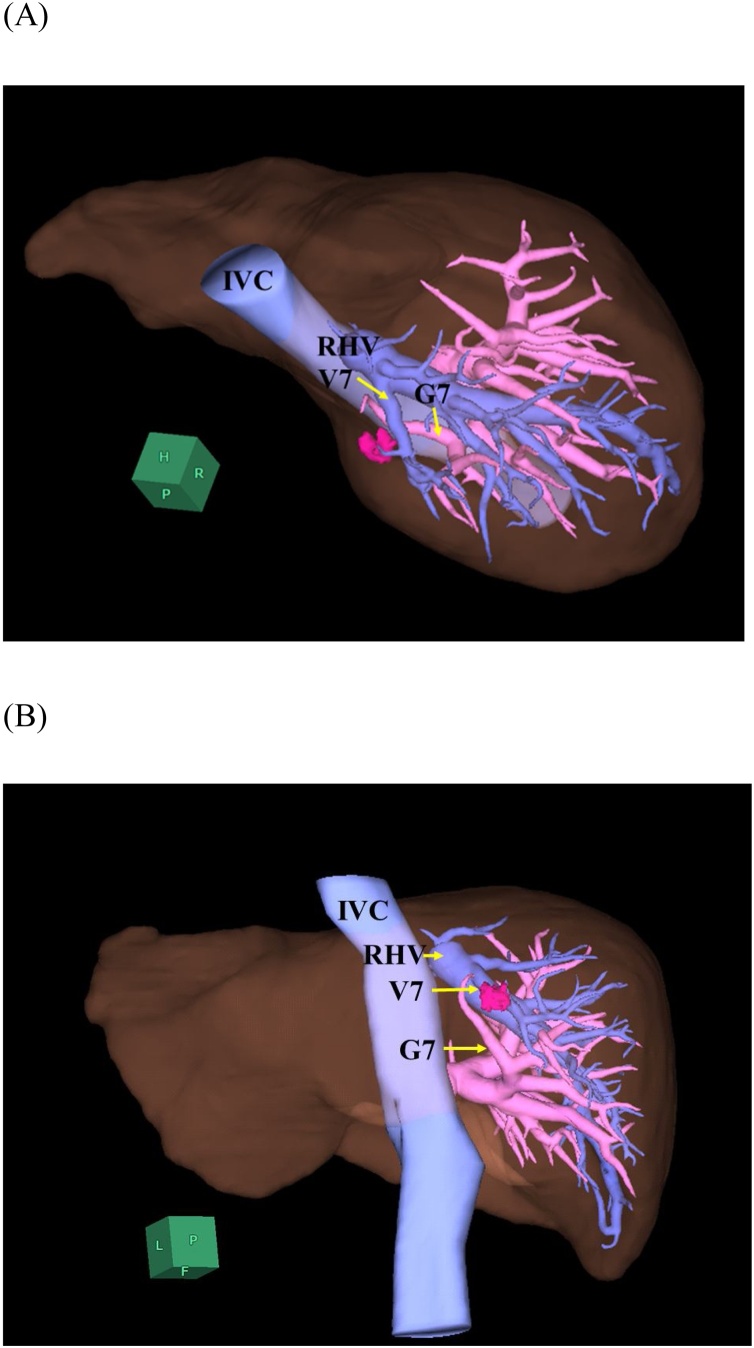
The three-dimensional imaging based on computed tomography showed colorectal liver metastasis, located at the top of segment 7 behind the right hepatic vein. IVC, inferior vena cava; RHV, right hepatic vein; G7, Glissonean pedicle of segment 7; and V7, venous branch of segment 7.

A 28-year-old female was referred to our hospital with the diagnosis of rectosigmoid cancer and synchronous liver metastases at the segment three (S3) and S7. The patient had no drug history, family history including any relevant genetic information, and psychosocial history. Following chemotherapy with five cycles of FOLFOX (5-fluorouracil and oxaliplatin) plus panitumumab, a partial response to liver metastases was identified by radiological imaging, showing the tumors measuring1cm in S3 and 1.5 cm in S7. Tumor in S7 was located at the top of S7 behind the right hepatic vein, and the relationship between the tumor, the Glissonean branch of S7 (G7) and the venous branch of S7 (V7) was depicted in Fig. 1. The patient was suffered from the side effects of chemotherapy, therefore laparoscopic simultaneous resection for colon and liver metastases was scheduled. Hepatic functional reserve revealed normal function with the Child-Pugh grade A (score 5) and indocyanine green (ICG) retention rate at 15 min of 3.4%.

**Fig. 2:**
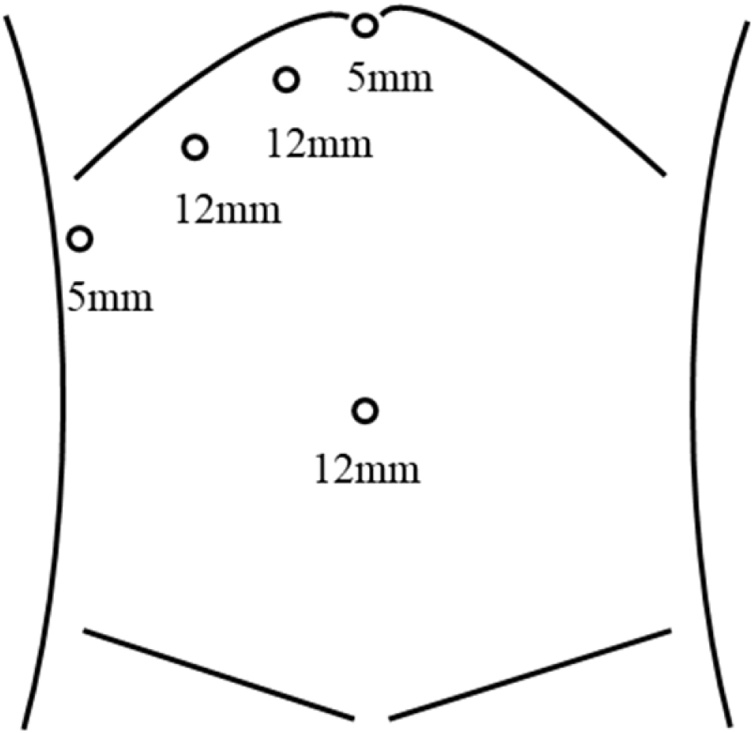
Trocar placement with the subcostal type.

**Fig. 3:**
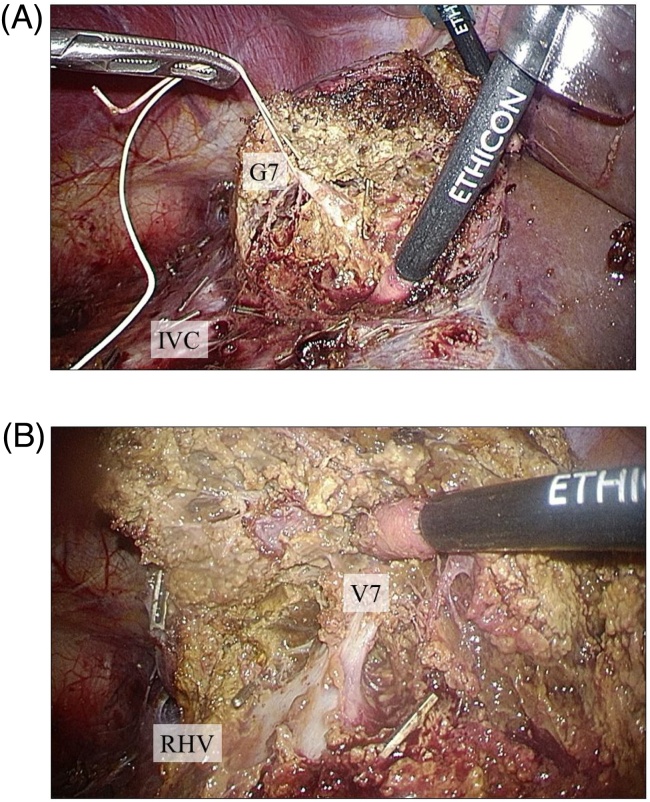
Intraoperative findings. (A) The Glissonean pedicle of segment 7 (G7) was identified and divided. (B) The right hepatic vein was exposed to identify the venous branch of segment 7 (V7).

Regarding the procedure, LLR was initially started. The patient was placed in the supine position with the operator (KT) at the right side and the assistant and scopist (TK and HA) at the left side. After the introduction of five trocars at the umbilical portion for the camera and at the subcostal area (Fig. 2), the left semi-decubitus position with the right side elevated approximately 20 degrees was applied. First the right lobe was completely mobilized with transection of a few short hepatic veins. Intraoperative ultrasound was used to confirm the tumor at S7, and the dissection line which secured a 2 cm margin from the tumor was decided. The liver parenchyma was dissected using the Cavitron Ultrasonic Surgical Aspirator (CUSA) and Ultrasonic shears (Harmonic scalpel), and the G7 was divided (Fig. 3A). The RHV was also exposed to transect the V7 (Fig. 3B). The parenchyma between RHV and dissection line was divided from the dorsal side, afterwards the specimen of S7 was removed (Supplementary Video 1). Following additional partial resection of S3, LLR for liver metastases was completed. Subsequently, laparoscopic low anterior resection with covering stoma for rectosigmoid cancer was performed by the colorectal surgery team. The total operative time was 506 min, including 180 min for LLR and 336 min for colorectal surgery. The estimated blood loss was minimal in total.

The postoperative course was uneventful with the patient being discharged on postoperative day 9. Pathological examination of the liver specimens confirmed colorectal metastases with free surgical margins.

## 3 Discussion

The present study presents our experience of LLR-S7 for the tumor located at the top of S7, successfully performed with the intrahepatic Glissonean approach. Several important points of view should be acknowledged to achieve safer approach to S7 by laparoscopy. Therefore a literature review was performed in order to summarize surgical techniques for LLR-S7.

Regarding patient positioning, the left semi-decubitus position with the right side elevated approximately from 30 to 45 degrees is mostly introduced [1,3]. In contrast, another option is the semi-prone position [5]. In our experience, the left semi-decubitus position was easier to introduce, and did not require to understand unfamiliar anatomical distortions.

**Fig. 4:**
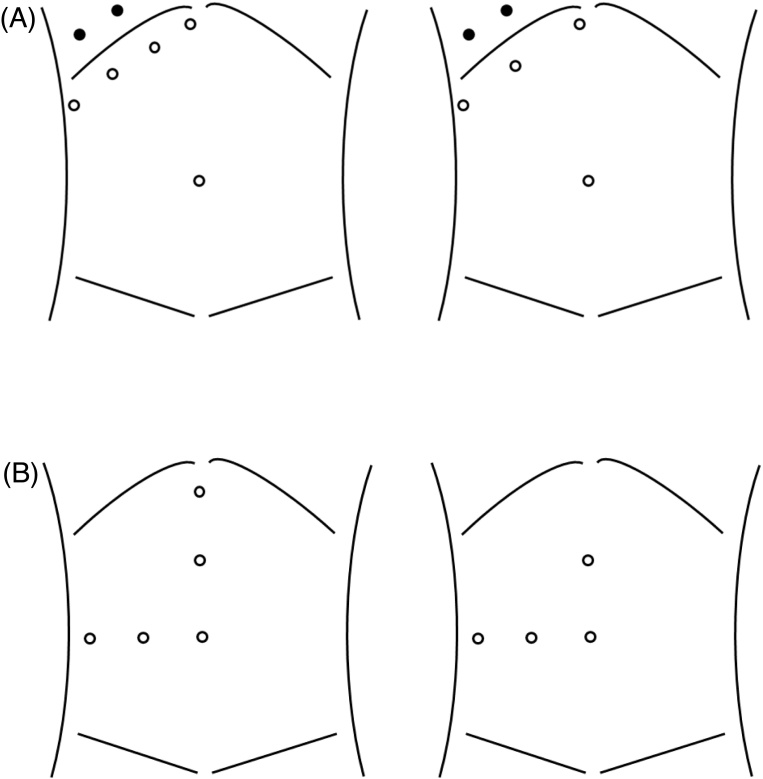
Trocar placement. (A) The subcostal type with four or five trocars techniques (circle). One or two intercostal trocars can be added if necessary (black circle). (B) The reverse L type with four or five trocars techniques (circle).

The trocar placement can be divided into two types, including the subcostal type or reverse L type (Fig. 4). In subcostal type, three or four trocars are placed along the lower edge of the right ribs. One or two intercostal trocars can be added if necessary. The use of intercostal trocars would help in a case with difficult mobilization of the right lobe or unclear visualization of the superior part of the liver. Inserting a laparoscope through an intercostal trocar can allow a direct view toward the RHV and IVC, and could avoid mutual interference of surgical instruments [2,6]. The advantages and feasibility of intercostal trocars on LLR-S7 have been demonstrated [2,6]. However, potential risks associated with intercostal trocars should be recognized. On the other hand, three or four trocars technique is available in the reverse L type. The incision for the Pringle maneuver is often placed on the left middle-upper abdomen. In our case, subcostal type without intercostal trocars was introduced. Actually, the complete mobilization of the right lobe was safely performed, and sufficient surgical field was obtained without mutual interference of surgical instruments using this method.

Various laparoscopic approaches for S7 have been reported so far. First, the Glissonian approach to the G7 from the liver hilum has been shown [7,8]. This technique requires incising the Rouviere sulcus and exposing the right posterior hepatic pedicle in order to identify the G7, therefore incidental biliary complications and bleeding might happen. More advanced laparoscopic skills and experiences are mandatory to use the Glissonian approach. ICG fluorescence imaging may be useful to recognize the parenchymal transection line [8]. Second, the intrahepatic Glissonean approach has been demonstrated [1,9]. In this approach, the parenchyma is divided above the IVC from the root of the right hepatic vein to identify the G7. Afterwards, the RHV is exposed from the root to the peripheral to avoid incidental injury of the RHV. This technique can eliminate the risk of biliary complications compared to the Glissonian approach which required the dissection around the liver hilum. However, the proper identification of the G7 might be difficult. Third, the caudate lobe first approach is suggested [5,10]. In this technique, the caudate lobe is first divided at the middle from the caudal side to detach the caudate process from the posterior Glissonean pedicle, and the G7 can be identified. Sufficient anatomical understanding would enable this approach to be performed. Finally, the lateral approach from intercostal ports is reported as described above [2,11]. Use of intercostal trocars can provide a clear visualization of the superior part of the liver, and might help reduce the operator’s stress. In the present case, we introduced the intrahepatic Glissonean approach since other approaches require additional dissection around the liver hilum and caudate process, which might cause biliary complications and bleeding.

With respect to retraction system, the advantage of rubber band retraction method and sling technique have been reported [3,12]. However, experiences should be required to use these methods successfully.

Several limitations should be disclosed regarding LLR-S7. The evidence of each approach for S7 was based on small number of experiences. Although different techniques have been demonstrated, no study was performed to compare outcomes between these techniques. Furthermore, long-term outcomes following LLR-S7 for cancers are still unknown.

## 4 Conclusion

We report our experience of LLR-S7 using the intrahepatic Glissonean approach. In addition, the present study demonstrates a literature review regarding various techniques for LLR-S7. LLR-S7 can be performed safely with advanced laparoscopic techniques and sufficient knowledge on various approaches for S7.

## Declaration of Competing Interest

The authors have no conflicts of interest to declare.

## Source of funding

Not applicable.

## Ethical approval

Because this was a single report, and not a trial or observational research, there was no requirement for ethical approval.

## Consent

Written informed consent was obtained from the patient for publication of this case report and accompanying images. A copy of the written consent is available for review by the Editor-in-Chief of this journal on request.

## Author contributions

All authors contributed to this work, and approved the final manuscript.

## Registration of research studies

Not applicable.

## Guarantor

Kosei Takagi.

## Provenance and peer review

Not commissioned, externally peer-reviewed.
